# Wage Disparities across Immigrant Generations: Education, Segregation, or Unequal Pay?

**DOI:** 10.1177/00197939241261688

**Published:** 2024-06-12

**Authors:** JooHee Han, Are Skeie Hermansen

**Affiliations:** * JooHee Han is Researcher in the Department of Sociology and Human Geography at the University of Oslo. Are Skeie Hermansen is Professor of Sociology in the Department of Sociology and Human Geography at the University of Oslo and Researcher in the Swedish Institute for Social Research at Stockholm University

**Keywords:** wages, immigration, within-job pay gap, assimilation, inequality, discrimination

## Abstract

Immigrants and their native-born children often face considerable wage penalties relative to natives, but less is known about whether this inequality arises through differences in educational qualifications, segregation across occupations and establishments, or unequal pay for the same work. Using linked employer–employee data from Norway, the authors ask whether immigrant–native wage disparities 1) reflect differences in detailed educational qualifications, labor market segregation, or within-job pay differences; 2) differ by immigrant generation; and 3) vary across different segments of the labor market. They find that immigrant–native wage disparities primarily reflect sorting into lower-paying jobs, and that wage disadvantages are considerably reduced across immigrant generations. When doing the same work for the same employer, immigrant-background workers, especially children of immigrants, earn similar wages to natives. Sorting into jobs seems more meritocratic for university graduates, for professionals, and in the public sector, but within-job pay differences are strikingly similar across market segments.

Immigrants from low-income origin countries and their native-born children of visible minority status often earn considerably less than observably similar natives (Heath and Cheung 2007; Duleep 2015). Yet, less is known about the relative degree to which wage disparities reflect immigrants and natives working in different workplaces and occupations or arise through immigrant–native differences in pay when doing the same work for the same employer as well as unequal treatment in occupational sorting at the point of hire and later promotion decisions. Clarifying the role of employers in creating the workplace inequalities faced by immigrant minorities should thus be a high research priority if we are to gain a better understanding of the proximate organizational mechanisms behind these wage disparities and devise effective policies to combat them (Bielby 2000; Reskin 2003).

In this study, we address several key questions using linked employer–employee administrative data covering the entire Norwegian labor market. Do immigrants earn similar wages and experience similar wage growth compared to native co-workers who do the same work for the same employer? Or do immigrant–native wage disparities arise through differential sorting into lower-paying occupations and workplaces, including initial job sorting upon hiring? How do wage differences compared to natives, as well as the relative contribution of within-job pay disparities and between-job segregation, change across immigrant generations?

Given our focus on identifying the role of employers, a key strength of our data is that we can assess immigrant–native differences in contractual hourly wage between employees who occupy the same job (i.e., same occupation and employer), which is the context in which differential treatment in wage-setting can happen. Further, we accurately control for observed human capital using a fine-grained measure of educational qualifications, distinguishing between more than 300 degrees (i.e., combining educational level and detailed field of study).

We make several contributions to the previous literature. First, our analysis provides novel insights on the role of employers in generating inequality by disentangling whether immigrant–native disparities in hourly wages and annual wage growth arise from employers’ differential pay by immigrant background or differential sorting of immigrant workers across occupations and workplaces. Earlier studies have shown that immigrants tend to concentrate in different workplaces than natives (Åslund and Skans 2010; Andersson et al. 2014; Glitz 2014; Lillehagen and Hermansen 2022), that between-workplace variation in pay is a key source of immigrants’ earnings disadvantages (Aydemir and Skuterud 2008; Pendakur and Woodcock 2010; Barth, Bratsberg, and Raaum 2012; Bartolucci 2014; Dostie, Li, Card, and Parent 2021; Åslund, Bratu, Lombardi, and Thoresson 2023), and that improved access to higher-paying firms is a central component in immigrants’ earnings catch-up relative to natives over time (Barth et al. 2012; Ansala, Åslund, and Sarvimäki 2021; Arellano-Bover and San 2023). This literature has yet to study immigrant–native wage disparities between co-workers doing the same work for the same employer, which is central to assessing the claim of differential pay by employers.

Second, we assess variation in wage disadvantages to natives by immigrant generation. Only a few prior studies using linked employer–employee data have focused on children of immigrants (Melzer, Tomaskovic-Devey, Schunck, and Jacobbinghaus 2018; Lillehagen and Hermansen 2022; Peters and Melzer 2022). In particular, Melzer et al. (2018) found, on average, only small wage penalties net of human capital among second-generation immigrants, but substantial variation in the immigrant–native wage gaps across workplaces. We distinguish between immigrants arriving as adults or during childhood and native-born children of immigrants, which enables examining more precisely how differences in wage disparities unfold across immigrant generations.

Third, we analyze how between-job sorting and within-job wage gaps differ across labor market segments, defined by skill levels, institutional sector, organizational size, and types of occupations. This investigation provides a comprehensive assessment of variation in immigrant–native wage gaps across parts of the economy assumed to differ in terms of meritocratic reward systems and bureaucratic regulation.

## Sources of Wage Disadvantage across Immigrant Generations

Many immigrants who come to high-income countries from lower-income countries earn low wages relative to natives upon arrival, and, although reduced with years in the host country, these disparities often persist over the migrant’s life course (Heath and Cheung 2007; Duleep 2015; Villarreal and Tamborini 2018; Rho and Sanders 2021). Although economic disadvantages seldom disappear in the second generation, immigrants’ native-born children often experience intergenerational progress in the labor market (Heath, Rothon, and Kilpi 2008; Drouhot and Nee 2019; Villarreal and Tamborini 2023).

Differences in wage disadvantages compared to natives across immigrant generations can arise through various mechanisms. A first reason why immigrants often face labor market difficulties is because country-specific human capital, such as formal qualifications and cultural and institutional know-how, often have limited international transferability (Chiswick and Miller 2009; Duleep 2015). Lack of proficiency in the dominant language in the host country is a central barrier to labor market success among foreign-born workers, especially for the newly arrived (McManus, Gould, and Welch 1983; Dustmann and Van Soest 2002; Chiswick and Miller 2009). Immigrants can also experience lower economic returns to educational degrees obtained abroad due to both lack of recognition and lower quality of schooling in less-developed nations (Kanas and Van Tubergen 2009; Lancee and Bol 2017; Li and Lu 2023; Drange, Helland, and Hermansen 2024). Sojourner orientations and plans of return migration can also lead immigrants to make different labor market investments than natives (Dustmann 1993).

By contrast, children of immigrants—born and raised in their parent’s country of destination—obtain native-level language proficiency and domestic educational degrees with similar quality and signaling value as those of natives (Heath et al. 2008; Drouhot and Nee 2019). According to standard supply-side explanations, productivity-related factors should be less relevant for ethnic inequalities in earnings and employment, net of education, in the native-born second generation. To analyze immigrant–native wage gaps in this study, we adjust for individual education and human capital using highly detailed measures of educational degrees that differentiate between levels of attainment and fields of study. If worker skills and formal qualifications are behind the immigrant–native wage disparities, detailed controls for educational degrees should account for most of the wage disadvantages observed among immigrants and, especially, native-born children of immigrants.

A second source of wage disparities between immigrants and natives is widespread ethnic segregation across occupations and workplaces that pay different wages. Skill-based labor market sorting may lead immigrant-background workers to find employment in lower-paying workplaces than natives, and research finds that firm-specific wage premia are increasingly important for labor market inequality (Song et al. 2019; Tomaskovic-Devey et al. 2020; Håkanson, Lindqvist, and Vlachos 2021). For immigrant co-workers, shared language skills likely reduce communication barriers, which may incentivize employers to hire foreign-born workers with similar language skills or national origins (Hellerstein and Neumark 2008; Andersson et al. 2014; Åslund, Hensvik, and Skans 2014; Glitz 2014).

Segregation of immigrants and natives across workplaces can also reflect ethnic inequality in social networks and access to job-related information (Granovetter 1995; Aguilera and Massey 2003; Waldinger and Lichter 2003). Immigrants often have fewer personal contacts with natives and use the social networks of immigrant neighbors to find work, and immigrant managers often recruit employees from within their own national origin group (Kanas, Chiswick, Van Der Lippe, and Van Tubergen 2012; Åslund et al. 2014; Goel and Lang 2019). Residential segregation and industry of employment also explain a large share of the excessive likelihood that immigrants and natives work for different employers (Åslund and Skans 2010; Andersson et al. 2014; Glitz 2014; Lillehagen and Hermansen 2022). If skill-based and network-based labor market sorting is a central process, immigrant–native wage disparities should be reduced when comparing workers within occupations, workplaces, and jobs.

A third source of immigrant–native wage disparities is employer discrimination at varying stages of organizational careers (Petersen and Saporta 2004; Fernandez and Fernandez-Mateo 2006; Bills, Di Stasio, and Gërxhani 2017). Discrimination refers to situations in which employers or their agents treat workers with the same qualifications and productivity unequally based on ascribed group membership because of preferences and prejudice, stereotypes, or unconscious biases (Altonji and Blank 1999). To begin, employers can discriminate at the point of hire either by not extending job offers to minority applicants or by sorting newly hired minority employees into a lower-paying job compared to similarly qualified natives (Petersen and Saporta 2004). Prior research shows that racialized phenotypic traits and ethnocultural markers, such as skin color, foreign-sounding names, and religious signals, matter and that hiring discrimination against immigrant-background ethnic minority workers is widespread in most countries and often extends beyond the first callback into later stages of the recruitment process (Quillian et al. 2019; Han 2020; Quillian, Lee, and Oliver 2020). Given our focus on the gainfully employed, the contribution of discrimination at the point of hire to immigrant–native wage disparities is observed conditional on hiring. These inequalities can arise through employers’ initial sorting of newly hired immigrant-background workers into lower-paying starting jobs than similarly qualified natives or, more generally, by restricting access to job offers in higher-paying workplaces. However, our observational data make it difficult to adequately distinguish allocative discrimination in hiring from unobserved skill-based sorting and network-based job matching.

Moreover, another mechanism through which immigrant–native wage disparities can arise is that employers pay different wages to employees with immigrant and native background who do the same work in the same workplace, that is, within-job pay discrimination (Petersen and Saporta 2004). For example, lack of information and other job-search frictions may reduce the bargaining power of less-mobile immigrant workers, which provides employers more latitude for pushing down immigrants’ wages through monopsonistic discrimination (Hirsch and Jahn 2015). Finally, immigrant–native wage differences can also accumulate if minority and majority employees who occupy the same (initial) positions experience systematic differences in wage growth and promotion rates over time.

As argued by Petersen and Saporta (2004), however, we have good reason to expect the scope for employer discrimination to be greatest during the hiring process (i.e., initial callbacks, offering the job to a given candidate, and the quality of these offers) because of the lack of transparent information for job applicants at this point. By contrast, discrimination in wage-setting and promotion decisions is more constrained because it is easier for victims of illegitimate unequal treatment based on ascriptive characteristics to assemble unambiguous evidence of discrimination.

If within-job immigrant–native differences in wage levels and annual wage growth are small or nonexistent, this implies that wage disparities arise through differential job sorting, which, in turn, can reflect either employers’ hiring discrimination or differences in the role of skills and networks in job-matching processes. If nontrivial within-job differences in wage levels and wage growth between immigrants and natives are present, however, it is difficult to disentangle the contribution of employer discrimination to these disparities from that of unobserved worker productivity (e.g., differences in language proficiency). For native-born children of immigrants, unobserved skill differences should be less likely to explain lower wages compared to natives with very similar educational degrees who do the same work for the same employer.

## Variation across the Labor Market: Meritocracy and Bureaucratization

Although the aforementioned mechanisms are of a general nature, other processes may be specific to certain segments of the labor market. According to neo-assimilation theory, a key issue is whether disadvantages related to ethnoracial minority status will dissipate once immigrant minorities gain access to higher-status jobs and workplaces in the mainstream economy (Drouhot and Nee 2019). Yet, whether ethnoracial distinctions matter less for economic opportunities in the well-regulated parts of the labor market, arguably characterized by more meritocratic reward structures and organizational bureaucratization, is an empirical question.

Stratification research has shown that the direct effects of socioeconomic origin on labor market outcomes tend to be lower among those with postsecondary degrees (Hout 1988; Mastekaasa 2011; Hällsten 2013). According to this meritocracy hypothesis, the labor market for college-educated professionals is more meritocratic because higher qualifications provide powerful signals to employers that leave less room for social networks and non-productive factors. Thus, immigrant–native pay gaps should be smaller among workers with college education and within professional occupations, which often require advanced degrees (i.e., master’s or PhD) (Drange et al. 2024). For example, a recent meta-analysis of 97 field experiments reports that ethnoracial hiring discrimination is, on average, lower among job applicants with postsecondary degrees compared to applicants with less education (Quillian et al. 2019). By contrast, other studies have found larger immigrant–native pay gaps in white-collar occupations (Tomaskovic-Devey, Hällsten, and Avent-Holt 2015; Heizmann, Busch-Heizmann, and Holst 2017), which could suggest that employers’ scope for discrimination in pay setting is larger in occupations in which individual productivity assessments and merit-based wage bargaining is more prevalent (Castilla and Benard 2010). Thus, we are agnostic as to whether immigrant–native pay gaps are higher or lower among college-educated workers, who are often found in professional occupations.

Discrimination by ethnic background is prohibited by Norwegian law, as in most other Western countries, but legal enforcement and compliance are likely to vary by organizational context (Heath, Liebig, and Simon 2013). The scope for illegitimate unequal treatment is likely to be more constrained if employers rely on formal rules and regulations when making decisions on hiring, promotions, and wage setting, which helps disadvantaged minorities including women and ethnoracial minorities (Reskin 2000). The public sector constitutes the classic Weberian ideal type of a bureaucratic organization (Weber [1922] 1978). More formalized personnel policies increase transparency and managerial accountability, and more centralized wage setting provides less scope for individual negotiation and employer discretion (Kalev, Dobbin, and Kelly 2006; Dobbin, Schrage, and Kalev 2015; Wilson and Roscigno 2016; Mandel and Semyonov 2021). Conversely, private firms are less likely to be subject to monitoring and less prone to use formalized rules and regulations in their organizational practice. Large private companies, however, often exhibit more bureaucratic organizational structures and more often adopt formalized employment procedures, which are likely to reduce the scope of discriminatory organizational processes (Marsden, Cook, and Kalleberg 1996). Moreover, prior research has shown that unionization and more centralized and standardized wage bargaining reduce immigrant–native wage differentials (Peters and Melzer 2022; Drange et al. 2024). According to these arguments, immigrant–native wage disparities should be lower in more bureaucratized organizational contexts. While this study is not able to identify bureaucratic regulations directly, we use the public versus private sector and employer size as indirect proxies of organizational formalization.

## Immigrants in the Norwegian Labor Market

We consider the labor market in Norway to be especially interesting because of the combination of an ethnically stratified workforce and a well-regulated labor market, with a large public sector, strong labor unions, and high levels of centralized wage determination. These institutional characteristics contribute to a compressed wage inequality and are assumed to equalize employment protection and remuneration of immigrant-background workers (Barth, Moene, and Willumsen 2014; OECD 2019; Drange et al. 2024). The centralization of wage bargaining agreements does, however, vary across the labor market segments. Centralization is most pronounced in blue-collar occupations in the private sector, and in large parts of the public sector, while wage determination among white-collar employees in the private sector is to a higher extent determined in local bargaining and based on individual merits. Moreover, the Norwegian welfare state provides a generous social safety net, free access to education, and a redistributive social insurance system (Barth et al. 2014). Measured by intergenerational earnings elasticity, Norway’s level of intergenerational mobility is among the highest globally (OECD 2018), which is conducive to upward mobility among children from low-income families, many with immigrant origins. Simultaneously, educational expansion has reduced the relative supply of native-born workers to low-skill routine occupations, which has increased employer demand for immigrants to fill low-status jobs in the secondary labor market (Friberg and Midtbøen 2019; Bratsberg, Moxnes, Raaum, and Ulltveit-Moe 2022; Østbakken, Orupabo, and Nadim 2023). Nonetheless, the well-regulated Norwegian economy is likely a conservative case with regard to employers’ scope for differential pay by immigrant background, as institutional constraints on discriminatory practices arguably are stronger than in less-regulated labor markets.

In terms of immigration, Norway is similar to the increasingly ethnically diverse countries in Western Europe, with a current population share of immigrants and their native-born children of approximately 20% (OECD 2022; Statistics Norway 2023). Non-European immigration to Norway began around 1970 with young, unskilled male labor migrants arriving from Pakistan, Turkey, and Morocco. In 1975, a change in immigration policies effectively ended low-skilled labor immigration from low-income countries but continued to allow for immigration according to three main principles, which are still largely in place: 1) demand for specific high-skilled labor; 2) entry of refugees and political asylum seekers; and 3) family-based immigration for kin of immigrants already in Norway (Brochmann and Kjeldstadli 2008). From about 1980, Norway has experienced successive waves of refugee immigration (e.g., from Vietnam, Chile, and Iran in the 1980s; the Balkans, Iraq, and Somalia in the 1990s and 2000s; and Syria in the mid-2010s). After the European Union enlargements in 2004 and 2007, labor migration from new Eastern European member states—primarily recruited for low-skilled manual work—surged due to the principle of free internal mobility between countries in the European Economic Area (Bratsberg et al. 2022). Eastern European labor migrants can start working upon arrival, whereas refugees need to complete an introductory training program to acquire basic language skills and a cultural-institutional understanding of Norwegian society.

Regardless of entry criteria, however, immigrants from Eastern Europe and low-income non-European countries often arrive with low levels of formal education, earn less, and over time experience declining employment rates and increased social insurance dependency compared to natives (Bratsberg, Raaum, and Røed 2014; Drange et al. 2024). Today, the labor market in Norway is highly segregated by ethnicity and low-skilled immigrants are disproportionately concentrated in industries dominated by manual and routine occupations, where wages often are low, such as construction, hotels, restaurants, cleaning, and care work (Friberg and Midtbøen 2019; Lillehagen and Hermansen 2022; Østbakken et al. 2023). Children of immigrants, by contrast, have the advantage of improved educational opportunities and often make considerable gains in earnings compared to their foreign-born parents, despite well-documented and persistent employer discrimination at the point of hire (Quillian et al. 2019; Lillehagen and Hermansen 2022; Borgen and Hermansen 2023). In sum, ethnic stratification in immigrant-dense, low-wage segments combined with strong labor market progress of the coming-of-age second generation makes the Norwegian welfare state economy an interesting case for studying organizational sources of immigrant–native wage inequality.

## Data and Methods

We use linked employer–employee administrative data that include all formal jobs and establishments covering the entire Norwegian labor market between 2016 and 2020. Using unique person and establishment identifiers, we match information on employers and employees across several population-wide administrative registers. This approach allows us to 1) compare employees working in the same occupation for the same employers, and to make such comparisons between immigrants, children of immigrants, and natives; 2) assess the role of detailed educational qualifications and labor market sorting; and 3) study wage growth across years. Following establishments and their employees from year to year, we have information on approximately 7.8 million person-year observations.

For each worker, we have information on contractual hours worked and monthly salary on contracted hours, which excludes wages on overtime, as well as occupation, establishment, and sector of employment. The wage data are based on records reported by the establishments, compiled by Statistics Norway, which are considered very reliable, and are used in centralized wage bargaining. Furthermore, we include information on each person’s and their parents’ country of birth, year of arrival for the foreign-born, detailed measures of educational degrees, seniority in the current workplace (number of years employed in the establishment), age, sex, marital status, number of children (below age 20), and the municipality of residence.

For our purposes, we restrict our analysis to prime-age workers of 25–50 years old, focusing only on the highest-paying job spell per year if a person had multiple employment relations. For immigrant-background workers, our focus is restricted to persons from Eastern Europe, Africa, Asia, and Latin America, since labor market disadvantages are primarily found among ethnoracially visible immigrant minorities from these world regions (Hermansen 2013; Bratsberg et al. 2014; Drange et al. 2024). Finally, we exclude a small number of observations with missing information on key variables used in the analysis. These restrictions yield an analytic sample of 2,037,107 persons. Table 1 presents descriptive statistics separately for adult immigrants (*n* = 285,402), childhood immigrants (*n* = 49,596), native-born children of immigrants (*n* = 20,666), and natives (*n* = 1,681,443).

**Table 1.** Summary Statistics of Variables Used in Analyses by Immigrant Background

	All	By immigrant background			
		Natives	Adult immigrants	Childhood immigrants	Children of immigrants
	(1)	(2)	(3)	(4)	(5)
Log hourly wage					
Mean	5.557	5.589	5.362	5.434	5.476
SD	0.314	0.311	0.266	0.281	0.300
Female (fraction)	0.478	0.484	0.447	0.445	0.480
Age					
Mean	40.182	40.514	39.515	34.452	31.427
SD	8.896	9.015	7.714	7.300	5.634
Married (fraction)	0.424	0.398	0.608	0.415	0.373
Number of children (fraction)					
No children	0.412	0.402	0.461	0.435	0.586
One child	0.215	0.214	0.226	0.200	0.162
Two children	0.267	0.275	0.217	0.244	0.174
Three+ children	0.107	0.109	0.095	0.121	0.078
Seniority					
Mean	5.226	5.503	3.595	4.131	3.502
SD	4.548	4.690	3.149	3.686	3.059
Educational attainment (fraction)					
No registered education	0.022	0.001	0.174	0.009	0.012
Less than upper-secondary education	0.156	0.142	0.223	0.294	0.187
Upper-secondary education	0.347	0.362	0.256	0.322	0.262
University degree, Bachelor	0.323	0.344	0.183	0.257	0.335
University degree, Master	0.140	0.141	0.133	0.112	0.199
University degree, PhD	0.012	0.009	0.030	0.005	0.004
Occupation (fraction)					
Managers	0.099	0.111	0.027	0.060	0.064
Professionals	0.291	0.315	0.133	0.214	0.294
Technicians and associate professionals	0.164	0.177	0.076	0.149	0.202
Clerical and service non-manual occupations	0.249	0.236	0.310	0.388	0.341
Skilled manual occupations	0.152	0.137	0.270	0.139	0.078
Unskilled manual occupations	0.044	0.024	0.184	0.050	0.020
Sector and establishment size (fraction)					
Small private establishments	0.416	0.406	0.489	0.429	0.399
Medium-sized private establishments	0.164	0.155	0.220	0.186	0.184
Large private establishments	0.032	0.032	0.027	0.039	0.058
Public sector	0.388	0.406	0.264	0.347	0.359
*n* persons	2,037,107	1,681,443	285,402	49,596	20,666
*n* person-years	7,802,129	6,622,400	941,870	170,000	67,859
*n* municipalities	480	480	480	480	480
*n* educational level-and-field groups	323	270	315	223	190
*n* occupations	401	400	392	370	341
*n* establishments	229,914	207,408	84,500	32,678	15,195
*n* occupation–establishment units	744,382	667,902	150,115	47,890	21,512

*Notes:* Author's own calculations based on administrative data provided by Statistics Norway. SD, standard deviation.

### Variable Measurements

From the contractual monthly salary and contractual hours worked, we calculate the hourly wage, paid on regular work hours excluding bonuses, in Norwegian kroner (NOK), then adjust it using the consumer price index to 2020 current wage and transform it as the natural logarithm. The contractual hourly wage does not conflate pay on regular and overtime hours and does not include bonuses. Since a key goal of our analysis is to assess whether there is differential pay by employers related to immigrant background, we focus on the rate of pay and avoid mixing this with the total hours worked and other sources of income. Although self-evident, it should be noted that our focus is restricted to workers in employment, and we can therefore not assess immigrant–native inequality in access to employment.^1^ In auxiliary analyses, we also measure the annual change in log hourly wages between two consecutive years (i.e., annual wage growth).

We measure immigrant background using information on the country of birth of each person and their parents.^2^ We distinguish between three main immigrant generational groups, with natives as a reference, which refers to all persons born in Norway or abroad with at least one Norwegian-born parent: 1) adult immigrants are persons who were born abroad with two foreign-born parents and arrived at age 18 or older; 2) childhood immigrants are persons who were born abroad with two foreign-born parents who immigrated before age 18; 3) children of immigrants are persons who were born in Norway from two foreign-born parents. We also report results from one analysis in which we differentiate between the 10 largest countries of origin—Pakistan, Vietnam, Turkey, India, Philippines, Morocco, Chile, Sri Lanka, Philippines, and Poland—in addition to a category for the remaining non-Western countries of origin in our main sample.

Education is measured using information on each person’s highest completed level and field of education, obtained from the Norwegian version of the International Standard Classification of Education (ISCED-97, see Statistics Norway 2001). We use two measures of educational qualifications: 1) educational attainment measured as the highest level of completed education, using the eight categories, ranging from primary education to doctoral degrees; and 2) detailed educational qualifications, differentiating between 323 degrees, measured using combined information on the educational attainment level, type of degree, and field of study (i.e., 3-digit level and field codes). A key limitation of our data is the nontrivial share of adult immigrants who lack information on education (17.4%), primarily those with educational degrees from abroad that have not been registered in Norway’s national register for education. Thus, both measures of education include a separate category for persons without registered education. For childhood immigrants (0.9%) and children of immigrants (1.2%), the share without registered education is low and on par with non-migrant natives. Thus, educational qualifications are better captured for childhood immigrants and children of immigrants. However, we obtain similar results for immigrants when we exclude persons without registered information on education and when we do not adjust for education (see Online Appendix Tables A.1–A.3 for these results and more detailed information on immigrants’ lack of registered education).

We use measures of occupations, establishments, and unique combinations of the two (i.e., jobs) to capture dimensions of labor market segregation. The occupational code we use is finely detailed, with 401 occupations based on 4-digit codes in Norway’s Standard Classification of Occupations (Statistics Norway 1998), which is the Norwegian implementation of the internationally comparable International Standard Classification of Occupations (ISCO). Establishments are our measures of workplaces, where individuals actually work, not the employing firms (except for single establishment firms), and we use data on 299,914 establishments. Occupation–establishment units, our measure of jobs, is the unique combination of occupations and establishments; we have 744,382 job units in our data. Thus, jobs capture positions where employees do the same, or very similar, work for the same employer. This approach follows the standard job definition in the literature, wherein jobs differ from occupations by referring to specific positions in a physical site or workplace whereas an occupation refers to a collection of jobs involving similar activities across establishments (Penner et al. 2023).^3^

To assess variation in immigrant–native wage disparities across labor market segments, we differentiate workers across various dimensions: educational attainment levels, types of occupations (managers; professionals; technicians and associate professionals; clerical and service non-manual occupations; skilled manual occupations; unskilled manual occupations), public versus private sector, and establishment size (small, less than 50 employees; medium-sized, 50 to 499 employees; large, 500 or more employees).

### Analysis Strategy

The aim of our analysis is to assess whether the immigrant–native wage gaps reflect differences in detailed educational degrees; unequal sorting across occupations, workplaces, and jobs; or within-job unequal pay. We assess how wage differences to natives vary between adult immigrants, childhood immigrants, and children of immigrants. The Online Appendix provides details on the equations used in the linear regression models described below.^4^

Focusing on differences in hourly wage levels, our main analysis reports results from a series of six linear regression specifications. We first present the result from a baseline model that includes immigrant background, a basic set of sociodemographic characteristics (sex, age, number of children, marital and civil status, workplace seniority, and municipality), and year of observation of each worker. In the second and third regressions, we adjust for educational attainment level and then replace this with our measure of detailed educational qualifications with fields of specialization. These first three models do not account for the occupations or establishments in which employees work.

Using standard panel data techniques (Petersen 2004; Wooldridge 2010), our next step is to exploit the multilevel structure of our data to assess the role of differential sorting across occupations, establishments, and jobs, that is, occupation–establishment units (Penner et al. 2023). In the fourth, fifth, and sixth regressions, we therefore successively add separate controls for occupation, establishment, and occupation–establishment units using fixed-effect models, interacted with the observation year. These fixed-effect models provide the weighted average of immigrant–native wage differences for workers within the same occupations, establishments, and jobs in the same year. Note that estimates from the occupation–establishment fixed-effect specifications address whether immigrant–native wage gaps are present when we compare employees doing the same work for the same employer. Comparing estimates across the various estimators shows where the education-adjusted immigrant–native wage inequalities arise, either from differential pay within the same job or from differential sorting across occupations, establishments, and jobs.

Next, we use the same model specifications with which we first restrict our focus to study newly hired employees (i.e., employees in their first year of employment in the current establishment). Second, we change our outcome to the annual change in hourly wages and restrict the sample to employees who remain in the same establishment across the two consecutive years. Together, these analyses allow us to assess whether the immigrant–native wage differences are observed between employees after the initial sorting into the first job after hiring and whether there are immigrant–native differences in annual wage growth among employees who remain in the same establishment (e.g., internal promotions). Finally, we assess variation in immigrant–native wage differences by country of origin and across the labor market segments defined by levels of educational attainment, types of occupations, and by sector and establishment size.

## Results

Table 2 summarizes the coefficients on wages of adult immigrants, childhood immigrants, and children of immigrants from the series of regressions. In panel A, our baseline model (column (1)) shows that wages of adult immigrants, childhood immigrants, and children of immigrants, respectively, are approximately 25%, 15%, and 9% lower than those of natives before adjusting for education. In the model adjusting for level of educational attainment (column (2))—which are the estimates comparable to most studies of immigrant–native wage gaps—these wage disadvantages are reduced to 21% for adult immigrants, 9% for childhood immigrants, and 6% for children of immigrants. In the next step, adjusting for the detailed educational qualification fixed effects (column (3)) reduces the wage disadvantage among immigrants to about 19%, suggesting that immigrants are found in lower-paying fields of education within a given educational attainment level. For childhood immigrants, the wage gap is still approximately 9% after adjusting for the educational qualification fixed effects, whereas controlling for detailed education yields a slightly larger wage disadvantage relative to natives—at about 8%—among children of immigrants.

**Table 2.** Estimated Immigrant–Native Gaps in the Logarithm of Hourly Wage for All Employees (Panel A), Logarithm of Hourly Wage for Newly Hired Employees (Panel B), and Annual Change in Logarithm of Hourly Wage for Employees Staying in the Same Establishment (Panel C)

	Basic adjustments	Education, level	Education, level and field	Within		
				Occupation	Establishment	Occupation–Establishment
	(1)	(2)	(3)	(4)	(5)	(6)
**Panel A. Log hourly wage of all employees**						
Adult immigrants	−0.248***	−0.213***	−0.188***	−0.097***	−0.125***	−0.056***
	(0.000)	(0.000)	(0.000)	(0.000)	(0.000)	(0.000)
Childhood immigrants	−0.147***	−0.086***	−0.086***	−0.043***	−0.041***	−0.015***
	(0.001)	(0.001)	(0.001)	(0.000)	(0.000)	(0.000)
Children of immigrants	−0.087***	−0.062***	−0.077***	−0.047***	−0.038***	−0.017***
	(0.001)	(0.001)	(0.001)	(0.001)	(0.001)	(0.001)
*R^2^*	0.275	0.430	0.470	0.628	0.631	0.778
Observations (person-years)	7,802,129	7,802,129	7,802,129	7,802,129	7,802,129	7,802,129
**Panel B. Log hourly wage of new employees**						
Adult immigrants	−0.258***	−0.220***	−0.197***	−0.099***	−0.120***	−0.054***
	(0.001)	(0.001)	(0.001)	(0.001)	(0.001)	(0.001)
Childhood immigrants	−0.136***	−0.077***	−0.076***	−0.034***	−0.029***	−0.008***
	(0.001)	(0.001)	(0.001)	(0.001)	(0.001)	(0.001)
Children of immigrants	−0.063***	−0.048***	−0.061***	−0.034***	−0.025***	−0.007***
	(0.002)	(0.002)	(0.002)	(0.002)	(0.002)	(0.002)
*R^2^*	0.250	0.400	0.439	0.611	0.631	0.763
Observations (person-years)	1,209,134	1,209,134	1,209,134	1,209,134	1,209,134	1,209,134
**Panel C. Annual change in log hourly wage**						
Adult immigrants	0.000	0.000	0.001*	0.002***	0.002***	0.001**
	(0.000)	(0.000)	(0.000)	(0.000)	(0.000)	(0.000)
Childhood immigrants	−0.004***	−0.003***	−0.002***	−0.001**	−0.001*	−0.001*
	(0.000)	(0.000)	(0.000)	(0.000)	(0.000)	(0.001)
Children of immigrants	−0.002*	−0.001	−0.001†	−0.001	−0.001	−0.001
	(0.001)	(0.001)	(0.001)	(0.001)	(0.001)	(0.001)
*R^2^*	0.011	0.011	0.013	0.017	0.115	0.119
Observations (person-years)	3,560,932	3,560,932	3,560,932	3,560,932	3,560,932	3,560,932

*Notes:* Panel A reports estimates from ordinary least squares (OLS) regressions in which immigrant background was regressed on log hourly wages for the period 2016–2020. Panel B reports estimates from OLS regressions in which immigrant background was regressed on log hourly wages for workers in the first year of employment in the current establishment in the period 2017–2020, since we do not observe workplace seniority in the year prior to 2016 for all workers. Panel C reports estimates from OLS regressions in which immigrant background was regressed on the annual change in log hourly wages for the period 2017–2020. Since 2016 is the first year with employment data, the first year with analysis of wage changes is from 2016 to 2017. The wage change analyses were restricted to employees who stayed in the same establishment between two adjacent years. The basic adjustments model (column (1)) controls for sex, marital status, number of children, age and age squared, seniority and seniority squared, year of observation, and municipality of residence, while the next two models successively add controls for eight levels of educational attainment (column (2)) and detailed educational level-and-field fixed effects (column (3)). Adjusting for basic controls and detailed educational level-and-field fixed effects, the next three models separately control for occupation (column (4)), establishment (column (5)), and occupation–establishment (column (6)) fixed effects. Huber-White heteroskedasticity robust standard errors are clustered on the person.

* *p* <0.05; **p <0.01; ***p <0.001.

How do these immigrant–native wage gaps change as we successively adjust for sorting across occupations (column (4)), establishments (column (5)), and occupation–establishment units (column (6))? The patterns are qualitatively similar in all immigrant generations. Taking sorting into occupations and establishments into account reduces the immigrant–native wage gaps considerably. The within-occupation and within-establishment wages are 10–13% lower than those of natives for adult immigrants and 4–5% lower for childhood immigrants and children of immigrants. Controlling for occupation–establishment further reduces the estimated immigrant–native wage gaps. The within-job wages of adult immigrants are 6% lower than natives, while they are less than 2% for childhood immigrants and children of immigrants.

The wage disadvantages for adult immigrants, childhood immigrants, and children of immigrants are largely attributable to differences in formal educational qualifications and sorting into lower-paying occupations, establishments, and jobs compared to natives. For immigrant-background workers, within-job wage differentials make up 11–23% of the total wage disadvantages before adjustment for education. After taking differences in detailed education into account, the within-job wage gaps make up 31% (adult immigrants), 18% (childhood immigrants), and 23% (children of immigrants) of the immigrant–native wage gaps (i.e., comparing the estimates in columns (3) and (6)), while the remaining gap is attributable to sorting on occupations, establishments, and occupation–establishment units.

Although immigrants who arrived as adults still earn less than natives when they work alongside each other, we find it difficult to exclude the possibility that the remaining residual gaps reflect unobserved productivity differences such as language skills. However, childhood immigrants and, in particular, children of immigrants, whose productivity-related traits net of education are likely to be less relevant, earn practically the same wages as natives when doing the same work for the same employer. This finding suggests that within-job differential pay is likely to be a minor source of wage disadvantages among immigrant-background workers, but employer discrimination can of course arise through channeling them into lower-paid jobs at the point of hire or in later promotions.

### Immigrant Disadvantages in Starting Wages and Annual Wage Growth within Workplaces?

The above analyses show that the substantial wage disadvantages for immigrant-background employees are only to a limited degree driven by differential pay for the same work, especially for childhood immigrants and children of immigrants. To the extent that immigrant-background workers’ wage disadvantages reflect employers’ differential treatment, these gaps are likely to arise through sorting into different job positions at the point of hiring or through differences in later promotion rates. Because of lack of information on applicant pools, we are unable to study the hiring process as such, but we can assess immigrant–native differences in starting wages in the initial job after hiring and differentials in annual wage growth.

In Table 2, panel B, we report coefficients of immigrant–native wage gaps when limiting our focus to differences between newly hired employees, which allows us to assess whether immigrant–native differences in pay are present in the first year after recruitment. The estimated gaps in wage levels are relatively similar to those reported for all immigrant generations in the models in panel A. In particular, when comparing newly hired employees in the establishment fixed effect model (column (5)), adult immigrants, childhood immigrants, and children of immigrants earn approximately 12.0%, 2.9%, and 2.5%, respectively, less than observably similar natives, net of detailed control for educational qualifications, in the same workplace. This outcome suggests that a nontrivial part of the wage disparities results from immigrants being sorted into lower-paying jobs than comparable native co-workers hired by the same employer, which is also true, but less pronounced, for childhood immigrants and children of immigrants. Turning to the within-job differences (column (6)), we again observe small differentials in pay once we compare childhood immigrants and children of immigrants to native co-workers in the same job. For childhood immigrants and children of immigrants, note that the within-establishment and within-job gaps for newly hired employees are about one percentage point smaller than those reported for all employees (panel A). The sample of newly hired employees is, however, different from and considerably smaller than the sample of all employees, which complicates the interpretation of the substantively small differences between the two panels.

Table 2, panel C, presents coefficients for immigrant–native differences in annual changes in wages for employees who remained in the same establishment across two adjacent years. The estimates show that the gaps in wage growth experienced by adult immigrants, childhood immigrants, and children of immigrants relative to natives are close to zero in all model specifications and often also not statistically significant. In particular, this finding is also true when comparing annual wage growth of employees with immigrant and native backgrounds who occupied the same job in the first of the two years when wage growth is compared.

On the whole, these results suggest that the wage disadvantages do not primarily reflect within-job wage differences or wage growth within workplaces, but rather arise through differential sorting into occupations and establishments during the job search process or through allocative discrimination at the point of hire (i.e., both in terms of not being offered a job with higher-paying employers and through differential sorting into lower-paying jobs after being hired). In the following analyses, we focus on differences in the levels of hourly wages and address whether heterogeneity is present in immigrant–native gaps between different national ancestry groups and segments of the labor market.

### Differences in Immigrant–Native Wage Inequality across Countries of Origin

**Figure 1.**
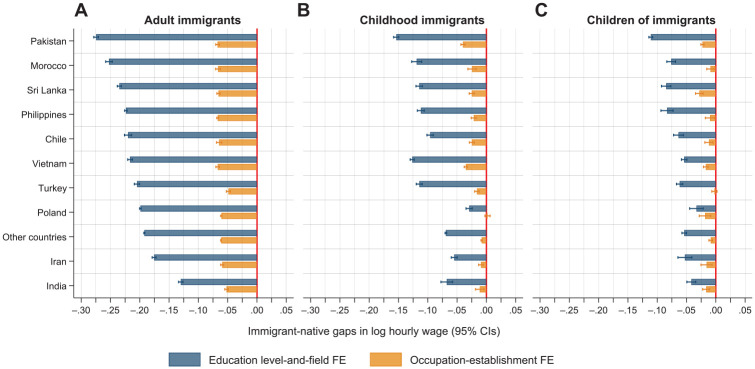
Estimated Immigrant–Native Gaps in Hourly Wages by Country of Origin from OLS Regressions (95% Confidence Intervals) *Notes:* The education level-and-field fixed effect model (top, blue bar) controls for sex, marital status, number of children, age and age squared, seniority and seniority squared, year of observation, municipality of residence, and educational level-and-field fixed effects. The occupation–establishment fixed effect model (bottom, orange bar) additionally controls for occupation–establishment fixed effects. The Huber-White heteroskedasticity robust standard errors are clustered on the person. CIs, confidence intervals; FE, fixed effects; OLS, ordinary least squares.

We continue by examining the variation in the wage gaps by country of origin. Figure 1 summarizes immigrant–native wage gaps separately for adult immigrants, childhood immigrants, and children of immigrants in 10 major national origin groups and the remaining origin countries. We present the estimated coefficients for the detailed educational qualification model (top, blue bars) and the occupation–establishment model (bottom, orange bars). The origin countries are sorted by the size of the education-adjusted wage disadvantages among adult immigrants.^5^

For adult immigrants, we report considerable variation in the size of the wage disadvantage relative to natives after adjusting for detailed education across origin countries. The largest gaps are found among immigrants from Pakistan, Morocco, and Sri Lanka, while the smallest gaps are found for immigrants from Iran and India. Controlling for sorting into occupation–establishment units strongly reduces the immigrant–native gaps for all national origin groups with the within-job wage gaps hovering between 5 and 7% in all groups.

Although we observe considerable variation in the size of the wage penalties, there is a strong pattern of assimilation toward natives across immigrant generations. Education-adjusted immigrant–native wage disparities among children of immigrants are less than half—often considerably more—of those found among adult immigrants within all national origin groups, with childhood immigrants falling in between in most cases. Childhood immigrants and children of immigrants from Pakistan experience the largest gaps of 11–15% after adjusting for detailed education measures. Childhood immigrants of Moroccan, Sri Lankan, Turkish, Vietnamese, Chilean, and Philippine origin have 10–12.5% lower earnings than equally qualified natives after adjusting for detailed education, while the corresponding gaps range from 5–8% for children of immigrants from the same origin countries. Both childhood immigrants and children of immigrants with Iranian, Indian, other non-Western, and, especially, Polish origins experience relatively small disadvantages net of educational controls.

Sorting into lower-paying jobs accounts for the remaining wage penalties almost entirely, even for the origin countries with the largest immigrant–native wage gaps net of education. For childhood immigrants, the within-job pay gaps are below 5% for all national origins, while within-job pay differentials are less than 2.5% and often close to zero in all origin groups among children of immigrants. Thus, Figure 1 supports the conclusion from the main analysis that immigrant–native wage gaps mainly reflect differences in education and sorting, whereas within-job pay gaps tend to be small both in relative terms and in substantive magnitudes.

### Differences in Immigrant–Native Wage Inequality across Labor Market Segments

**Figure 2.**
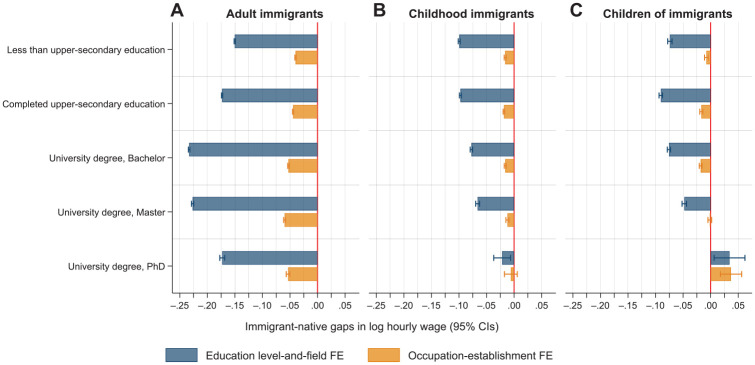
Estimated Immigrant–Native Gaps in Hourly Wages by Levels of Educational Attainment from OLS Regressions (95% Confidence Intervals) *Notes:* The education level-and-field fixed effect model (top, blue bar) controls for sex, marital status, number of children, age and age squared, seniority and seniority squared, year of observation, municipality of residence, and educational level-and-field fixed effects. The occupation–establishment fixed effect model (bottom, orange bar) additionally controls for occupation–establishment fixed effects. The Huber-White heteroskedasticity robust standard errors are clustered on the person. CIs, confidence intervals; FE, fixed effects; OLS, ordinary least squares.

In the following analyses, we assess variation in the immigrant–native wage gaps across segments of the labor market. Figure 2 reports the immigrant–native wage gaps estimated separately for various levels of educational attainment. For adult immigrants, the wage disparities relative to natives are largest among workers with bachelor’s and master’s degrees, while smaller gaps are found among workers with upper-secondary education or less, and doctoral degrees. However, the gaps are to a great extent explained by sorting across occupation–establishment units, and within-job wage gaps are approximately 5% at all levels of educational attainment.

For childhood immigrants and children of immigrants, the magnitude of the gaps is considerably smaller at all levels of educational attainment and, in contrast to adult immigrants, those with advanced degrees (i.e., master’s and PhD degrees) face the smallest education-adjusted wage gaps relative to natives. Except for graduates with PhD degrees, childhood immigrants and children of immigrants earn 5–10% less than natives. When doing the same work for the same employer we observe practically no wage differences relative to natives at either educational attainment level.

**Figure 3.**
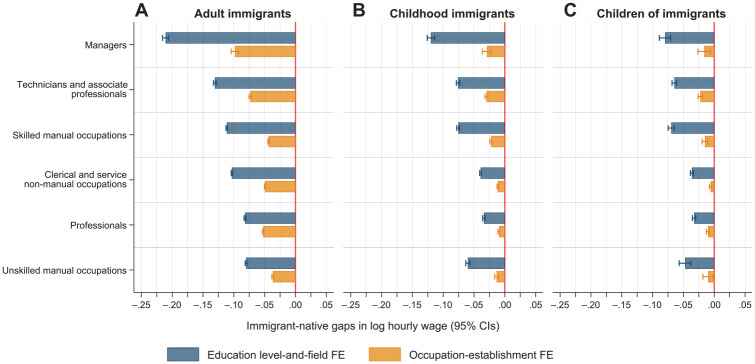
Estimated Immigrant–Native Gaps in Hourly Wages by Groups of Occupations from OLS Regressions (95% Confidence Intervals) *Notes:* The education level-and-field fixed effect model (top, blue bar) controls for sex, marital status, number of children, age and age squared, seniority and seniority squared, year of observation, municipality of residence, and educational level-and-field fixed effects. The occupation–establishment fixed effect model (bottom, orange bar) additionally controls for occupation–establishment fixed effects. The Huber-White heteroskedasticity robust standard errors are clustered on the person. CIs, confidence intervals; FE, fixed effects; OLS, ordinary least squares.

Next, we assess whether systematic variation exists in immigrant–native wage gaps across types of occupations. For adult immigrants, Figure 3 shows that the wage disadvantage relative to natives is the largest among managers (21%) and technicians and associate professionals (13%) after adjusting for detailed education. Wages are 10–11% below natives for immigrants in skilled manual occupations and clerical and service non-manual occupations, and among technicians and associate professionals after adjusting for education. The smallest gaps are found for professionals and unskilled manual workers, of whom adult immigrants earn 7–8% less than natives when adjusting for education. After adjusting for sorting across occupation–establishment units, the within-job disadvantages are approximately 5% in all occupations, except for managers and technicians and associate professionals, for whom the within-job gaps are about 9% and 7%, respectively. Although the size of the education-adjusted wage gaps is considerably smaller among childhood immigrants and children of immigrants, the pattern of variation between occupations is comparable to that found for immigrants, and in professional and clerical and service non-manual occupations the education-adjusted wage differences relative to natives are below 5%. Nonetheless, it should be noted that children of immigrants still face an earnings disadvantage of more than 5% even after accounting for detailed educational qualifications in many occupational groups. However, these gaps reflect differential sorting into occupation–establishment units, and the within-job wage differentials among children of immigrants are small and virtually non-existent among professionals and employees in clerical and service non-manual occupations.

Note that highly educated adult immigrants face substantial wage disadvantages, while those who work in professional occupations—that require advanced degrees—experience the lowest wage gaps. One likely explanation for this pattern is that many well-educated immigrants experience occupational downgrading relative to their educational credentials (Akresh 2008; Larsen, Rogne, and Birkelund 2018), while those who gain access to professional occupations experience a meritocratic reward structure. This outcome is consistent with prior research showing that licensure—which is common in many professional occupations—reduces within-occupation immigrant–native wage gaps (Drange et al. 2024). For childhood immigrants and children of immigrants, whom we should expect to experience more similar occupational returns to their educational credentials compared to natives, we observe consistent patterns of small or no wage gaps among both those with advanced university degrees (master’s and PhD) and those who work in professional occupations. These patterns suggest that the labor market for high-skilled professionals is more meritocratic for immigrant-background workers than are other parts of the economy.

**Figure 4.**
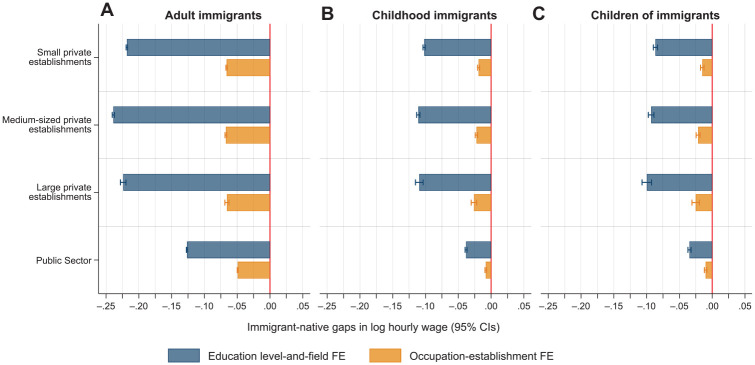
Estimated Immigrant–Native Gaps in Hourly Wages by Sector and Establishment Size in Private Sector from OLS Regressions (95% Confidence Intervals) *Notes:* The education level-and-field fixed effect model (top, blue bar) controls for sex, marital status, number of children, age and age squared, seniority and seniority squared, year of observation, municipality of residence, and educational level-and-field fixed effects. The occupation–establishment fixed effect model (bottom, orange bar) additionally controls for occupation–establishment fixed effects. The Huber-White heteroskedasticity robust standard errors are clustered on the person. CIs, confidence intervals; FE, fixed effects; OLS, ordinary least squares.

To explore the bureaucracy and organizational formalization hypothesis, Figure 4 reports sector differences in immigrant–native wage gaps when dividing private-sector workplaces based on establishment size (small, medium, and large). For adult immigrants, the largest education-adjusted immigrant–native gaps are found among employees in private-sector workplaces (21–23%) whereas immigrants’ wage disadvantages are considerably smaller in the public sector (12.5%). Adjusting for sorting into occupation–establishment units, however, reduces the gaps considerably across all types of establishments. The within-job wage gap is smallest in the public sector (5%) and approximately 6–7% for workplaces in the private sector regardless of size.

For childhood immigrants and children of immigrants, we find small immigrant–native wage gaps across all model specifications in the public sector. For private-sector workplaces, there is not much variation by establishment size after adjusting for education. Adjusting for sorting into occupation–establishment units reduces the immigrant–native wage gaps to 1–2.5% in all types of establishments. Although the within-job gaps are smallest in public workplaces, the difference relative to the private sector is small.

Overall, our results show that adult immigrants, childhood immigrants, and children of immigrants all face considerably smaller wage gaps, net of detailed education, in the public sector. We observe less systematic variation by establishment size in the private sector. However, when comparing workers in the same jobs we find only small variation in the immigrant–native wage gaps across sectors and workplaces of varying size. Thus, the larger immigrant–native wage gaps in the private sector seem primarily to reflect differences in within-sector sorting across occupations and establishments, whereas the differences in sorting net of education are smaller in the public sector.

## Conclusion and Discussion

Successful labor market incorporation of immigrants and their native-born children is of increasing importance in high-income host countries. To improve policies aimed at promoting equal opportunities it is crucial to identify sources of existing immigrant–native wage gaps. Using economy-wide administrative data from Norway, we examine whether immigrant–native wage disparities arise from unequal pay for the same job by employers; differential sorting into occupations, establishments, and jobs; or differences in educational qualifications. We assess how the magnitude and sources of immigrant–native wage inequality differ by immigrant generation and country of origin. We also address how immigrant–native wage inequality varies across segments in the labor market often assumed to differ according to meritocratic remuneration criteria and bureaucratic regulation.

Our key finding is that wage disparities between immigrant and native workers in the Norwegian labor market arise primarily from processes related to the acquisition of education and differential sorting in the labor market. Our results show that unequal pay for the same work and differential promotion rates are not the major sources of the wage disparities experienced by immigrant-background workers compared to natives. Segregation across occupations, establishments, and jobs likely reflects either, on the one hand, mechanisms related to skill-based sorting, job search behavior, and lack of access to natives’ social networks or, on the other hand, employer discrimination at the point of hire.

These findings resonate well with previous research concluding that sorting across firms and workplaces is the key source of immigrants’ earnings disadvantages relative to natives (Aydemir and Skuterud 2008; Barth et al. 2012; Dostie et al. 2021; Arellano-Bover and San 2023; Åslund et al. 2023). Our focus on within-job wage inequality across immigrant generations, however, allows us to address the role of employers’ unequal pay for the same work, which we find to be of limited importance for childhood immigrants and children of immigrants. Although differential pay or promotion rates by employers do not seem to contribute much to the overall wage disadvantages that immigrant-background workers face, ethnic discrimination against minority job applicants at the point of hire is well-established from field experiments (Quillian et al. 2019). Thus, we emphasize that the systematic sorting of immigrant-background workers into lower-paying positions in the labor market plausibly reflects, at least partly, employer hiring discrimination (Barth et al. 2012). Substantial variation in wage disadvantages, net of detailed educational controls, between phenotypically similar immigrant minorities, such as children of Pakistani, Iranian, and Indian immigrants, do, however, highlight the complexity of these sorting processes.

Despite nontrivial remaining wage disadvantages, we do find strong intergenerational economic progress when comparing children of immigrants and childhood immigrants to adult immigrants within all national ancestry groups. Consistent with previous research (Hermansen 2016; Lillehagen and Hermansen 2022), this study documents improved economic opportunities among children of immigrants but also highlights remaining barriers to equal access to higher-paying jobs. Nonetheless, the wage assimilation seen in the native-born second generation suggests that equalizing features of the Norwegian welfare state, such as its open educational system, overrides the inequality-generating contribution of employer discrimination and other non-meritocratic obstacles that children of immigrants often face in the labor market.

Our analyses of heterogeneity across labor market segments show a striking pattern of similarity in the size of within-job pay differentials across levels of educational attainment, types of education, and sector and organizational size within each immigrant generation. Thus, employers’ scope for unequal pay for the same work by immigrant background does not vary systematically across market segments. Thus, while other studies have found that within-workplace immigrant–native pay gaps vary with organizational demographic characteristics (Tomaskovic-Devey et al. 2015; Melzer et al. 2018; Peters and Melzer 2022), our analysis finds little evidence for within-job pay disadvantages among children of immigrants and immigrants who arrived as children in various segments of the Norwegian labor market. However, sorting into equally remunerated jobs for workers with similar education varies across different parts of the economy. For immigrant-background workers with advanced university degrees, at least for children of immigrants, and for those working in professional occupations and the public sector, the allocation to jobs seems to be the most meritocratic. This finding implies that both policies aimed at supporting the job-matching process as well as policy measures limiting employers’ possibilities for indulging in discriminatory recruitment practices have the potential to equalize economic opportunities for immigrants and their native-born offspring.

To conclude, the central contribution of our study is to establish the relative contribution processes relating to labor market sorting versus within-job pay disparities, as well as better identification of the organizational mechanisms through which immigrant–native wage inequality arises. Our findings show that immigrant–native differences in within-job pay and wage growth are modest in Norway, while differential sorting into lower-paying jobs at the point of hire may still constitute a key bottleneck that generates wage disadvantages among immigrant-background workers. This finding aligns well with research claiming that the opportunity structure for discrimination is largest during the hiring processes, whereas the room for employer discretion is often smaller when setting wages and making promotion decisions for workers doing the same work (Petersen and Saporta 2004). Although a vast literature reports on audit studies on hiring discrimination against immigrant-background workers (Quillian et al. 2019), there is another stream of research on gender and racial hiring disadvantages that use data on entire pools of applicants to specific job openings (Petersen, Saporta, and Seidel 2000; Fernandez and Fernandez-Mateo 2006). Extending this literature to studies of the recruitment processes of immigrants and their native-born children could provide important new knowledge to understand the relative role of supply-side and demand-side factors for hiring outcomes. Clearly, too, more research is needed on ethnic stratification in the organizational careers of immigrants and their native-born children, starting with the quality of the job offers employers extend to newly hired employees to the dynamics of wage-setting as well as promotions and advancement into higher-level positions. Because well-regulated economies, such as the one in Norway, may reduce the scope for employer discrimination in the workplace, a key task for future studies is to assess these questions across a broader and more institutionally diverse set of labor market contexts.

## Supplemental Material

sj-pdf-1-ilr-10.1177_00197939241261688 – Supplemental material for Wage Disparities across Immigrant Generations: Education, Segregation, or Unequal Pay? Supplemental material, sj-pdf-1-ilr-10.1177_00197939241261688 for Wage Disparities across Immigrant Generations: Education, Segregation, or Unequal Pay? by JooHee Han and Are Skeie Hermansen in ILR Review

## Footnotes

1 Limiting the sample to only individuals in employment introduces selection bias if we are interested in total wage inequality since immigrant-background workers often experience ethnic discrimination in hiring and other barriers in access to employment.

2 For children of immigrant parents born in different countries of origin, we use the information on the mother’s country of birth. Note that country of origin does not capture ethnicity as such, as this would require additional information on religion, culture, phenotype, and the like, which will vary within origin countries.

3 Online Appendix Table A.4 reports results when occupations and jobs (i.e., occupation–establishment units) are defined using 1-, 2-, and 3-digit occupational codes. The results show that within-job estimates of immigrant–native wage differences do not change substantively depending on the level of occupational granularity used to define jobs.

4 Our main analysis is pooled by gender, but separate analyses by gender reported in the Online Appendix show that the main conclusions are similar for men (Table A.5) and women (Table A.6). However, the immigrant–native wage differences tend to be larger among men, except at the within-job level.

5 Online Appendix Table A.7 reports immigrant–native wage gaps for Western European and North American origin countries, who are not included in our analytic sample. These results reveal considerably smaller wage disadvantages to natives among Western immigrants compared to immigrants from all other non-Western countries of origin.
