# Supplementary material for: Wage Disparities across Immigrant Generations: Education, Segregation, or Unequal Pay?
Source: Ind Labor Relat Rev. 2024 Jun 12;77(4):598–625. doi: 10.1177/00197939241261688 (PMC11305947; doi:10.1177/00197939241261688)
Supplement: sj-pdf-1-ilr-10.1177_00197939241261688 – Supplemental material for Wage Disparities across Immigrant Generations: Education, Segregation, or Unequal Pay? [file sj-pdf-1-ilr-10.1177_00197939241261688.pdf]

Online Appendices are the responsibility of the author(s);
the material is not copyedited or formatted by the *ILR Review*.

Online Appendix for

Wage Disparities across Immigrant Generations: Education, Segregation, or Unequal Pay?

JooHee Han and Are Skeie Hermansen

Corresponding author: Are Skeie Hermansen,
e-mail: a.s.hermansen@sosgeo.uio.no

Statistical models

We estimate a series of Ordinary Least Squares (OLS) regression models using six different model specifications which allow us to assess the degree to which immigrant–native wage differences to natives reflect (a) differences in detailed education, (b) differential sorting into occupations, establishments, and jobs (occupation-establishment units), and (c) within-job wage differences in wages among adult immigrants, childhood immigrants, and native-born children of immigrants.

In Table 2 (Panel A), the first model only adjusts for basic covariates (Model 1). This model includes covariate controls for gender, age, age squared, and geographic region of employment within the host country. The next model introduces controls for levels of educational attainment (Model 2) and then we replace this measure with controls for detailed combinations of levels of educational attainment and fields of study (Model 3). In the following three models we introduce fixed effects that allow us to compare adult immigrants, childhood immigrants, and children of immigrants to natives who work in the same occupation (Model 4), the same establishment (Model 5), and the same job (i.e., occupation–establishment unit; Model 6). The equations estimated for our six models follow the same general form, using six different specifications:

$$\ln \text{hourly wage}_{i,t} = \theta_{BASE}\boldsymbol{x}_{i,t} + \delta_t + \varepsilon_{i,t}, \tag{1}$$

$$\ln \text{hourly wage}_{i,t} = \theta_{EDULVL}\boldsymbol{x}_{i,t} + \beta_{EDULVL}edulevel_{i,t} + \delta_t + \varepsilon_{i,t}, \tag{2}$$

$$\ln \text{hourly wage}_{i,t} = \theta_{EDUFE}\boldsymbol{x}_{i,t} + \gamma_{edufe} + \delta_t + \varepsilon_{i,t}, \tag{3}$$

$$\ln \text{hourly wage}_{i,t} = \theta_{OCC}\boldsymbol{x}_{i,t} + \eta_{occ,t} + \gamma_{edufe} + \delta_t + \varepsilon_{i,t}, \tag{4}$$

$$\ln \text{hourly wage}_{i,t} = \theta_{EST}\boldsymbol{x}_i, t + \eta_{est,t} + \gamma_{edufe} + \delta_t + \varepsilon_{i,t}, \tag{5}$$

$$\ln \text{hourly wage}_{i,t} = \theta_{OCCEST}\boldsymbol{x}_{i,t} + \eta_{occest,t} + \gamma_{edufe} + \delta_t + \varepsilon_{i,t}, \tag{6}$$

where the subscripts represent *i* for individuals, *t* for years, *edufe* for detailed educational field of study, *occ* for occupations, *est* for establishments, and *occest* for occupation–establishment units. The dependent variable is the logarithm of the hourly wage (ln hourly wage$_i$) for individual *i*, and the independent variables are collected in the vector $\boldsymbol{x}_i$, which includes a constant; the gender, age, and age-squared of individual *i*; and a series of indicator variables for immigrant background, and geographic region of individual *i* in year *t*. The variable $edulevel_{i,t}$ measures the educational level of individual *i* in year *t*. The fixed effect γ_{edufe} refers to an indicator of all unique combinations of levels of educational attainment and detailed fields of study. The fixed effects $\eta_{\text{occ,t}}$, $\eta_{\text{est,t}}$, and $\eta_{\text{occest,t}}$ refer to fixed effects for occupation, establishment, and occupation–establishment units, respectively, interacted with year t (i.e., a separate fixed effect for each unit in each year). Our measure of immigrant background refers to an

indicator of native workers, the reference category, adult immigrants, childhood immigrants, and children of immigrants.

Model 1 thus provides estimates of the immigrant–native differences in earnings after basic adjustments for gender, age, age-squared, and geographic region. Model 2 adds controls for educational attainment and Model 3 replaces this control with a fixed effect for detailed educational field. Model 4 includes these same covariates as well as the fixed effects $\eta_{occ,t}$ representing the occupation indicators. Thus, model 4 provides estimates of immigrant–native differences in earnings obtained from comparing adult immigrants, childhood immigrants, and children of immigrants to natives who work in the same occupation in the same year. Intuitively, these results can be thought of as estimating the immigrant–native difference in wages separately for each occupation unit in each year and then taking a weighted average of these immigrant–native differences across all occupations by years. Models 5 and 6 are analogous to model 4, but contain the fixed effects $\eta_{est,t}$, and $\eta_{occest,t}$ that refer to the unique occupation by year ($\eta_{occ,t}$), establishment by year ($\eta_{est,t}$), or occupation–establishment by year ($\eta_{occest,t}$) unit. The subscripts to the θ parameters and β parameter indicate that these are different coefficients, pertaining to different model specifications.

In Panel B of Table 2, we estimate the identical model specifications but restricting our sample only to individuals observed in the first year of employment in the current establishment. The aim of this analysis is to assess wage differences between native and immigrant background coworkers in their first job in the establishment upon hiring. In this analysis, we restrict our sample to the years 2017–2020 since we do not want to include the first year of observation.

In Panel C of Table 2, we estimate the identical model specifications but change our outcome variable to the yearly change of the natural logarithm of hourly wages. The aim of this analysis is to assess differences in annual wage growth between native and immigrant background coworkers. In this analysis, we also exclude data from 2016 since we need to study wage changes from one year to the next and we also restrict our sample to employees who remain employed in the same establishment across to consecutive years.

In the remaining Figures 1–4, we present estimates for levels of log hourly wages separately by country of origin, level of educational attainment, type of occupation, and establishment sector and size. These estimates are based on model specifications which are identical to Model 3 (i.e., controlling for detailed educational field) and Model 6 (i.e., the within-job differences based on occupation–establishment by year fixed effects) described above.

Information on lack of registered education and sensitivity analyses

For the general population born in Norway, data on education comes directly from a number of sources including educational institutions and the governmental student loan agency. These data are updated annually and is updated for any given individual when a new educational degree has been completed. The quality, level of detail, and coverage is very good. However, Table 1 in the main text shows that information on education is missing for about 17.4% of the

adult immigrants (arriving at age 18 or older) in our sample. However, Table 1 shows that only about 1% of childhood immigrants (arriving at age 17 or younger) and native-born children of immigrants lack information on education. The main source of information on immigrants' education acquired abroad is self-reported education in the process of getting a residence permit (Norwegian Directorate for Immigration) as well as Statistics Norway's census surveys targeting the foreign-born population (in 1991, 1999, 2011, and 2012), information from accreditation of foreign educational degrees (Norwegian Agency for Quality Assurance in Education), and information provide when job seekers register their CVs in public databases (Norwegian Labour and Welfare Administration).

Table A.1 provides more detailed information on how the full distribution of educational levels, including those with missing information, vary among immigrants according to country of origin, year of immigration, wage quartiles, and occupations (1-digit ISCO occupational codes). For country of origin, the share of individuals with no registered information on education ranges from 7–8% (Philippines, Iran, and Sri Lanka) to 27% for Poland, followed by Vietnam (18%) and Pakistan, Vietnam, and Morocco (14–15%). For year of immigration, the share with missing information on education is highest for recently arrived immigrants, with 27% of immigrants arriving in 2010 or later and 8% among immigrants who arrived between 2000 and 2009. For immigrants arriving before year 2000, the share with missing information on education is 2–3%. For wage quartiles, missing information on education is most common among those in the first (lowest) wage quartile (20%) and second wage quartile (16%), while the share with missing is 11–12% for those in the third and fourth (highest) quartile. For occupations, it is most common to lack information on education among workers in skilled and unskilled manual occupations, where this share ranges between 22–27%. For the remaining occupations, the share with missing education ranges between about 9 to 12%. Thus, missing information on education is more common among recently arrived immigrants with low wages in manual occupations, where immigrants from Poland are overrepresented.

To test the sensitvitity of our estimates with regard to missing information on education, we, first, reestimate our models excluding workers with missing information (Table A.2) and, second, re-estimate our models not controlling for education in the within-occupation, within-establishment, and within-job models (Table A.3). Overall, these analyses show that our conclusions are robust to these tests, although the gaps are

Table A.2 shows that the first set of both models with and without adjustment of educational attainment and detailed educational field (columns 1–3) are very similat to those reported in the main analyses. This is also the case for the three models estimating the immigrant–native wage differences within occupations, establishments, and jobs.

Table A.3 shows a similar pattern and here we only present estimates for the basic adjustments (column 1), within-occupation (column 2), within-establishment (column 3), and within-job (column 4) models. For adult immigrants and native-born children of immigrants, the estimates are very similar to those reported in the main analysis for all models. For childhood immigrants,

the wage differences to natives are slightly larger in the within-occupation and within-establishment models but for the within-job estimates the differences are very small.

Occupational granularity and immigrant–native within-job differences

We follow standard conventions from the literature on within-job pay gaps in referring to the within occupation–establishment unit estimate as the "within-job" estimate. When conceptualizing jobs at the intersection of occupations and establishments, the aim is to compare coworkers who do particular tasks for the same employer. It is, however, not obvious what level of occupational granularity is most appropriate when defining jobs, as very detailed job titles may distinguish more between specific wage levels than differences in work tasks to be performed.

Table A.4 shows that the estimates at the within-job level are very similar when we compare models where occupation–establishment units are defined using four-digit occupational codes to models where the occupation–establishment units are defined at the one-, two-, and three-digit level. The results show that within-job estimates of immigrant–native wage differences do not change substantially depending on the level of occupational granularity used to define jobs. For example, in panel A the largest difference in the within-job estimates when comparing models using four-digit (column 2) to one-digit (column 4) occupational codes is found for adult immigrants and the difference is 0.011 log points (i.e., about 1 percentage point). Thus, our conclusions about the size of within-job immigrant–native wage differences remain broadly the same also when using coarsened job definitions.

Immigrant–native wage differences separately for men and women

There are many reasons that male and female immigrants and children of immigrants could face different obstacles in the labor market, which can produce systematic gender differences in immigrant–native pay gaps. Table A.5 and Table A.6 report estimates corresponding to Table 2 in the main text separately for men and women.

Comparing the estimates in the two tables, shows that men face notably larger immigrant–native wage disadvantages to natives compared to women in all three generational categories. However, the within-job differences in pay are similar for both men and women. This shows that sorting into lower-paying jobs makes an even larger contribution to the wage disadvantages experienced by immigrant-background male workers, but that they, like women, experience small gaps relative to natives when working in the same occupation for the same employer.

Immigrant–native wage differences for Western immigrants

Table A.7 report estimates corresponding to Table 2 in the main text for adult immigrants, childhood immigrants, and children of immigrants from origin countries in Western Europe and North America. These results show that immigrant-background workers from Western origin countries experience considerably smaller wage disadvantages to natives compared to the corresponding estimates for all non-Western immigrants combined (Table 1) and also all of the largest non-Western origin countries (Figure 1).

Table A.1. Summary statistics of variables used in analyses by immigrant background.

	Educational attainment					
	Missing	Less than upper-secondary education	Completed upper-secondary education	University degree, Bachelor	University degree, Master	University degree, PhD
	(1)	(2)	(3)	(4)	(5)	(6)
**Panel A. Country of origin**						
Poland	0.269	0.124	0.366	0.117	0.109	0.015
Turkey	0.116	0.393	0.264	0.136	0.066	0.026
Morocco	0.142	0.351	0.245	0.191	0.056	0.015
Sri Lanka	0.076	0.397	0.278	0.180	0.052	0.018
Philippines	0.070	0.169	0.243	0.387	0.116	0.015
India	0.180	0.093	0.070	0.302	0.247	0.108
Iran	0.078	0.198	0.192	0.254	0.200	0.079
Pakistan	0.148	0.346	0.129	0.202	0.146	0.029
Vietnam	0.146	0.408	0.222	0.137	0.062	0.026
Chile	0.092	0.201	0.318	0.256	0.102	0.030
Other countries	0.146	0.268	0.214	0.192	0.146	0.034
**Panel B. Year of arrival**						
Before 1990	0.031	0.279	0.330	0.267	0.079	0.014
1990-1999	0.024	0.289	0.327	0.244	0.097	0.019
2000-2009	0.082	0.256	0.310	0.195	0.130	0.027
2000-2019	0.266	0.188	0.206	0.162	0.143	0.035
**Panel C. Wage quartiles**						
First quartile (lowest)	0.204	0.303	0.256	0.156	0.072	0.009
Second quartile	0.156	0.163	0.360	0.194	0.114	0.014
Third quartile	0.114	0.100	0.208	0.280	0.228	0.069
Fourth quartile (highest)	0.116	0.045	0.073	0.202	0.423	0.141
**Panel D. Occupations**						
Managers	0.124	0.176	0.228	0.214	0.222	0.036
Professionals	0.086	0.035	0.036	0.320	0.389	0.134
Technicians and associate professionals	0.113	0.095	0.129	0.286	0.318	0.059
Clerical and service non-manual occupations	0.116	0.272	0.293	0.209	0.097	0.012
Skilled manual occupations	0.273	0.228	0.363	0.091	0.040	0.006
Unskilled manual occupations	0.223	0.329	0.252	0.131	0.057	0.009

Note: Author's own calculations based on administrative data provided by Statistics Norway.

Table A.2. Estimated immigrant—native gaps in logarithm of hourly wage for all employees (Panel A), logarithm of hourly wage for newly hired employees (Panel C), and annual change in logarithm of hourly wage for employees staying in the same establishment (Panel C) excluding all employees with no registered information on education. OLS regressions.

				Within		
	Basic adjustments	Education, level	Education, level and field	Occupation	Establishment	Occupation-Establishment
	(1)	(2)	(3)	(4)	(5)	(6)
**Panel A. Log Hourly Wage of All Employees**						
Adult immigrants	-0.243***	-0.215***	-0.191***	-0.100***	-0.129***	-0.059***
	(0.000)	(0.000)	(0.000)	(0.000)	(0.000)	(0.000)
Childhood immigrants	-0.146***	-0.085***	-0.085***	-0.043***	-0.041***	-0.015***
	(0.001)	(0.001)	(0.001)	(0.000)	(0.000)	(0.000)
Children of immigrants	-0.085***	-0.060***	-0.075***	-0.046***	-0.037***	-0.016***
	(0.001)	(0.001)	(0.001)	(0.001)	(0.001)	(0.001)
R^2	0.269	0.428	0.469	0.626	0.628	0.776
Observations (person-years)	7,626,613	7,626,613	7,626,613	7,626,613	7,626,613	7,626,613
**Panel B. Log Hourly Wage of New Employees**						
Adult immigrants	-0.257***	-0.257***	-0.257***	-0.096***	-0.127***	-0.048***
	(0.001)	(0.001)	(0.001)	(0.001)	(0.001)	(0.001)
Childhood immigrants	-0.136***	-0.136***	-0.136***	-0.045***	-0.048***	-0.012***
	(0.001)	(0.001)	(0.001)	(0.001)	(0.001)	(0.001)
Children of immigrants	-0.060***	-0.060***	-0.060***	-0.036***	-0.013***	-0.008***
	(0.002)	(0.002)	(0.002)	(0.002)	(0.002)	(0.002)
R^2	0.245	0.245	0.245	0.586	0.558	0.756
Observations (person-years)	1,160,252	1,160,252	1,160,252	1,160,252	1,160,252	1,160,252
**Panel C. Annual Change in Log Hourly Wage**						
Adult immigrants	0.000	0.000	0.001*	0.002***	0.002***	0.001**
	(0.000)	(0.000)	(0.000)	(0.000)	(0.000)	(0.000)
Childhood immigrants	-0.004***	-0.003***	-0.002***	-0.002**	-0.001*	-0.001*
	(0.000)	(0.000)	(0.000)	(0.000)	(0.000)	(0.001)
Children of immigrants	-0.002*	-0.001	-0.001	-0.001	-0.001	-0.001
	(0.001)	(0.001)	(0.001)	(0.001)	(0.001)	(0.001)
R^2	0.011	0.012	0.013	0.017	0.115	0.119
Observations (person-years)	3,490,426	3,490,426	3,490,426	3,490,426	3,490,426	3,490,426

Note: Panel A reports estimates from OLS regressions where immigrant background was regressed on log hourly wages for the period 2016-2020. Panel B reports estimates from OLS regressions where immigrant background was regressed on log hourly wages for workers in the first year of employment in the current establishment in the period 2017-2020, since we do not observe workplace seniority in the year prior to 2016 for all workers. Panel C reports estimates from OLS regressions where immigrant background was regressed on the annual change in log hourly wages for the period 2017-2020. Since 2016 is the first year with employment data, the first year with analysis of wage changes is from 2016 to 2017. The wage change analyses were restricted to employees who stayed in the same establishment between two adjacent years. The basic adjustments model (column 1) control for sex, marital status, number of children, age and age squared, seniority and seniority squared, year of observation, and municipality of residence, while the next two models successively add control for control for eight levels of educational attainment (column 2) and detailed educational level-and-field fixed effects (column 3). Adjusting for basic controls and detailed educational level-and-field fixed effects, the next three models separately control for occupation (column 4), establishment (column 5), and occupation-establishment (column 6) fixed effects. Huber-White heteroskedasticity robust standard errors are clustered on the person. All observations with no registered information on education are excluded.

* P<0.05, ** P<0.01, *** P<0.001

Table A.3. Estimated immigrant--native gaps in logarithm of hourly wage for all employees (Panel A), logarithm of hourly wage for newly hired employees (Panel C), and annual change in logarithm of hourly wage for employees staying in the same establishment (Panel C) without adjustment for educational qualifications. OLS regressions.

	Basic adjustments	Within: Occupation	Within: Establishment	Within: Occupation-Establishment
	(1)	(2)	(3)	(4)
**Panel A. Log Hourly Wage of All Employees**				
Adult immigrants	-0.248***	-0.095***	-0.137***	-0.051***
	(0.000)	(0.000)	(0.000)	(0.000)
Childhood immigrants	-0.147***	-0.054***	-0.064***	-0.019***
	(0.001)	(0.000)	(0.001)	(0.000)
Children of immigrants	-0.087***	-0.050***	-0.032***	-0.017***
	(0.001)	(0.001)	(0.001)	(0.001)
R^2	0.275	0.599	0.545	0.769
Observations (person-years)	7,802,129	7,802,129	7,802,129	7,802,129
**Panel B. Log Hourly Wage of New Employees**				
Adult immigrants	-0.258***	-0.094***	-0.125***	-0.045***
	(0.001)	(0.001)	(0.001)	(0.001)
Childhood immigrants	-0.136***	-0.045***	-0.048***	-0.012***
	(0.001)	(0.001)	(0.001)	(0.001)
Children of immigrants	-0.063***	-0.037***	-0.014***	-0.008***
	(0.002)	(0.002)	(0.002)	(0.002)
R^2	0.25	0.588	0.565	0.758
Observations (person-years)	1,209,134	1,209,134	1,209,134	1,209,134
**Panel C. Annual Change in Log Hourly Wage**				
Adult immigrants	0.000	0.003***	0.002***	0.002***
	(0.000)	(0.000)	(0.000)	(0.000)
Childhood immigrants	-0.004***	-0.002***	-0.002***	-0.001**
	(0.000)	(0.000)	(0.000)	(0.001)
Children of immigrants	-0.002*	-0.001	-0.001	-0.001
	(0.001)	(0.001)	(0.001)	(0.001)
R^2	0.011	0.016	0.115	0.120
Observations (person-years)	3,560,932	3,560,932	3,560,932	3,560,932

Note: Panel A reports estimates from OLS regressions where immigrant background was regressed on log hourly wages for the period 2016-2020. Panel B reports estimates from OLS regressions where immigrant background was regressed on log hourly wages for workers in the first year of employment in the current establishment in the period 2017-2020, since we do not observe workplace seniority in the year prior to 2016 for all workers. Panel C reports estimates from OLS regressions where immigrant background was regressed on the annual change in log hourly wages for the period 2017-2020. Since 2016 is the first year with employment data, the first year with analysis of wage changes is from 2016 to 2017. The wage change analyses were restricted to employees who stayed in the same establishment between two adjacent years. The basic adjustments model (column 1) control for sex, marital status, number of children, age and age squared, seniority and seniority squared, year of observation, and municipality of residence. The next three models separately control for occupation (column 2), establishment (column 3), and occupation-establishment (column 4) fixed effects. Huber-White heteroskedasticity robust standard errors are clustered on the person.

* $P<0.05$, ** $P<0.01$, *** $P<0.001$

Table A.4. Estimated immigrant--native gaps in logarithm of hourly wage for all employees (Panel A), logarithm of hourly wage for newly hired employees (Panel C), and annual change in logarithm of hourly wage for employees staying in the same establishment (Panel C) with occupation and occupation-establishment fixed effects defined at different levels of occupational granularity. OLS regressions.

	Main analysis		Sensitivity analyses of occupational granularity					
	Within (4-digit occupation)		Within (1-digit occupation)		Within (2-digit occupation)		Within (3-digit occupation)	
	Occupation	Occupation-Establishment	Occupation	Occupation-Establishment	Occupation	Occupation-Establishment	Occupation	Occupation-Establishment
	(1)	(2)	(3)	(4)	(5)	(6)	(7)	(8)
**Panel A. Log Hourly Wage of All Employees**								
Adult immigrants	-0.097***	-0.056***	-0.120***	-0.067***	-0.116***	-0.063***	-0.108***	-0.061***
	(0.000)	(0.000)	(0.000)	(0.000)	(0.000)	(0.000)	(0.000)	(0.000)
Childhood immigrants	-0.043***	-0.015***	-0.061***	-0.020***	-0.055***	-0.018***	-0.049***	-0.017***
	(0.000)	(0.000)	(0.000)	(0.000)	(0.000)	(0.000)	(0.000)	(0.000)
Children of immigrants	-0.047***	-0.017***	-0.060***	-0.023***	-0.054***	-0.021***	-0.051***	-0.019***
	(0.001)	(0.001)	(0.001)	(0.001)	(0.001)	(0.001)	(0.001)	(0.001)
R^2	0.628	0.778	0.571	0.748	0.590	0.760	0.608	0.769
Observations (person-years)	7,802,129	7,802,129	7,802,129	7,802,129	7,802,129	7,802,129	7,802,129	7,802,129
**Panel B. Log Hourly Wage of New Employees**								
Adult immigrants	-0.099***	-0.054***	-0.123***	-0.062***	-0.116***	-0.057***	-0.107***	-0.056***
	(0.001)	(0.001)	(0.001)	(0.001)	(0.001)	(0.001)	(0.001)	(0.001)
Childhood immigrants	-0.034***	-0.008***	-0.052***	-0.011***	-0.046***	-0.010***	-0.039***	-0.009***
	(0.001)	(0.001)	(0.001)	(0.001)	(0.001)	(0.001)	(0.001)	(0.001)
Children of immigrants	-0.034***	-0.007***	-0.047***	-0.012***	-0.042***	-0.011***	-0.038***	-0.009***
	(0.002)	(0.002)	(0.002)	(0.002)	(0.002)	(0.002)	(0.002)	(0.002)
R^2	0.611	0.763	0.558	0.739	0.579	0.747	0.594	0.755
Observations (person-years)	1,209,134	1,209,134	1,209,134	1,209,134	1,209,134	1,209,134	1,209,134	1,209,134
**Panel C. Annual Change in Log Hourly Wage**								
Adult immigrants	0.002***	0.001**	0.001***	0.001**	0.001***	0.001***	0.002***	0.001**
	(0.000)	(0.000)	(0.000)	(0.000)	(0.000)	(0.000)	(0.000)	(0.000)
Childhood immigrants	-0.001**	-0.001*	-0.002***	-0.001*	-0.002***	-0.001*	-0.002***	-0.001*
	(0.000)	(0.001)	(0.000)	(0.000)	(0.000)	(0.001)	(0.000)	(0.001)
Children of immigrants	-0.001	-0.001	-0.002	-0.001	-0.001	-0.001	-0.001	-0.001
	(0.001)	(0.001)	(0.001)	(0.001)	(0.001)	(0.001)	(0.001)	(0.001)
R^2	0.017	0.119	0.013	0.12	0.014	0.118	0.015	0.119
Observations (person-years)	3,560,932	3,560,932	3,560,932	3,560,932	3,560,932	3,560,932	3,560,932	3,560,932

Note: Panel A reports estimates from OLS regressions where immigrant background was regressed on log hourly wages for the period 2016-2020. Panel B reports estimates from OLS regressions where immigrant background was regressed on log hourly wages for workers in the first year of employment in the current establishment in the period 2017-2020, since we do not observe workplace seniority in the year prior to 2016 for all workers. Panel C reports estimates from OLS regressions where immigrant background was regressed on the annual change in log hourly wages for the period 2017-2020. Since 2016 is the first year with employment data, the first year with analysis of wage changes is from 2016 to 2017. The wage change analyses were restricted to employees who stayed in the same establishment between two adjacent years. All models control for sex, marital status, number of children, age and age squared, seniority and seniority squared, year of observation, municipality of residence, and detailed educational level-and-field fixed effects. Huber-White heteroskedasticity robust standard errors are clustered on the person.

* P<0.05, ** P<0.01, *** P<0.001

Table A.5. Estimated immigrant--native gaps in logarithm of hourly wage for all employees (Panel A), logarithm of hourly wage for newly hired employees (Panel C), and annual change in logarithm of hourly wage for employees staying in the same establishment (Panel C) for men. OLS regressions.

				Within		
	Basic adjustments	Education, level	Education, level and field	Occupation	Establishment	Occupation-Establishment
	(1)	(2)	(3)	(4)	(5)	(6)
**Panel A. Log Hourly Wage of All Employees**						
Adult immigrants	-0.271***	-0.242***	-0.224***	-0.116***	-0.149***	-0.063***
	(0.000)	(0.000)	(0.001)	(0.000)	(0.001)	(0.000)
Childhood immigrants	-0.177***	-0.116***	-0.115***	-0.057***	-0.055***	-0.019***
	(0.001)	(0.001)	(0.001)	(0.001)	(0.001)	(0.001)
Children of immigrants	-0.104***	-0.082***	-0.095***	-0.057***	-0.041***	-0.017***
	(0.002)	(0.001)	(0.001)	(0.001)	(0.001)	(0.001)
R^2	0.281	0.408	0.443	0.609	0.626	0.778
Observations (person-years)	4,070,006	4,070,006	4,070,006	4,070,006	4,070,006	4,070,006
**Panel B. Log Hourly Wage of New Employees**						
Adult immigrants	-0.264***	-0.232***	-0.213***	-0.103***	-0.118***	-0.047***
	(0.001)	(0.001)	(0.001)	(0.001)	(0.001)	(0.001)
Childhood immigrants	-0.160***	-0.099***	-0.098***	-0.043***	-0.033***	-0.008***
	(0.002)	(0.002)	(0.002)	(0.002)	(0.002)	(0.002)
Children of immigrants	-0.086***	-0.069***	-0.079***	-0.044***	-0.030***	-0.008**
	(0.003)	(0.003)	(0.003)	(0.002)	(0.003)	(0.003)
R^2	0.264	0.385	0.421	0.596	0.640	0.766
Observations (person-years)	624817	624817	624817	624817	624817	624817
**Panel C. Annual Change in Log Hourly Wage**						
Adult immigrants	-0.001***	-0.001***	-0.001**	0.001	-0.001**	-0.001**
	(0.000)	(0.000)	(0.000)	(0.000)	(0.000)	(0.001)
Childhood immigrants	-0.005***	-0.003***	-0.003***	-0.002**	-0.002***	-0.002*
	(0.001)	(0.001)	(0.001)	(0.001)	(0.001)	(0.001)
Children of immigrants	-0.001	-0.001	-0.001	0.000	-0.001	0.000
	(0.001)	(0.001)	(0.001)	(0.001)	(0.001)	(0.001)
R^2	0.009	0.01	0.011	0.015	0.132	0.138
Observations (person-years)	1874351	1874351	1874351	1874351	1874351	1874351

Note: Panel A reports estimates from OLS regressions where immigrant background was regressed on log hourly wages for the period 2016-2020. Panel B reports estimates from OLS regressions where immigrant background was regressed on log hourly wages for workers in the first year of employment in the current establishment in the period 2017-2020, since we do not observe workplace seniority in the year prior to 2016 for all workers. Panel C reports estimates from OLS regressions where immigrant background was regressed on the annual change in log hourly wages for the period 2017-2020. Since 2016 is the first year with employment data, the first year with analysis of wage changes is from 2016 to 2017. The wage change analyses were restricted to employees who stayed in the same establishment between two adjacent years. The basic adjustments model (column 1) control for sex, marital status, number of children, age and age squared, seniority and seniority squared, year of observation, and municipality of residence, while the next two models successively add control for control for eight levels of educational attainment (column 2) and detailed educational level-and-field fixed effects (column 3). Adjusting for basic controls and detailed educational level-and-field fixed effects, the next three models separately control for occupation (column 4), establishment (column 5), and occupation-establishment (column 6) fixed effects. Huber-White heteroskedasticity robust standard errors are clustered on the person.

* *P*<0.05, ** *P*<0.01, *** *P*<0.001

Table A.6. Estimated immigrant--native gaps in logarithm of hourly wage for all employees (Panel A), logarithm of hourly wage for newly hired employees (Panel C), and annual change in logarithm of hourly wage for employees staying in the same establishment (Panel C) for women. OLS regressions.

	Basic adjustments	Education, level	Education, level and field	Within: Occupation	Within: Establishment	Within: Occupation-Establishment
	(1)	(2)	(3)	(4)	(5)	(6)
**Panel A. Log Hourly Wage of All Employees**						
Adult immigrants	-0.217***	-0.180***	-0.155***	-0.082***	-0.107***	-0.052***
	(0.000)	(0.000)	(0.000)	(0.000)	(0.000)	(0.000)
Childhood immigrants	-0.111***	-0.049***	-0.049***	-0.024***	-0.026***	-0.010***
	(0.001)	(0.001)	(0.001)	(0.001)	(0.001)	(0.001)
Children of immigrants	-0.069***	-0.039***	-0.053***	-0.035***	-0.033***	-0.015***
	(0.001)	(0.001)	(0.001)	(0.001)	(0.001)	(0.001)
R^2	0.233	0.442	0.489	0.638	0.633	0.764
Observations (person-years)	3,732,122	3,732,122	3,732,122	3,732,122	3,732,122	3,732,122
**Panel B. Log Hourly Wage of New Employees**						
Adult immigrants	-0.245***	-0.203***	-0.176***	-0.094***	-0.114***	-0.058***
	(0.001)	(0.001)	(0.001)	(0.001)	(0.001)	(0.001)
Childhood immigrants	-0.105***	-0.048***	-0.050***	-0.024***	-0.024***	-0.007***
	(0.002)	(0.002)	(0.002)	(0.001)	(0.002)	(0.002)
Children of immigrants	-0.038***	-0.024***	-0.042***	-0.025***	-0.021***	-0.008**
	(0.003)	(0.003)	(0.002)	(0.002)	(0.003)	(0.002)
R^2	0.221	0.419	0.461	0.628	0.629	0.756
Observations (person-years)	584,314	584,314	584,314	584,314	584,314	584,314
**Panel C. Annual Change in Log Hourly Wage**						
Adult immigrants	0.002***	0.002***	0.002***	0.003***	0.004***	0.003***
	(0.000)	(0.000)	(0.000)	(0.000)	(0.000)	(0.000)
Childhood immigrants	-0.003***	-0.002*	-0.001	-0.001	0.000	0.000
	(0.001)	(0.001)	(0.001)	(0.001)	(0.001)	(0.001)
Children of immigrants	-0.002*	-0.002	-0.002	-0.001	-0.002	-0.002
	(0.001)	(0.001)	(0.001)	(0.001)	(0.001)	(0.001)
R^2	0.013	0.014	0.015	0.020	0.087	0.091
Observations (person-years)	1,686,580	1,686,580	1,686,580	1,686,580	1,686,580	1,686,580

Note: Panel A reports estimates from OLS regressions where immigrant background was regressed on log hourly wages for the period 2016-2020. Panel B reports estimates from OLS regressions where immigrant background was regressed on log hourly wages for workers in the first year of employment in the current establishment in the period 2017-2020, since we do not observe workplace seniority in the year prior to 2016 for all workers. Panel C reports estimates from OLS regressions where immigrant background was regressed on the annual change in log hourly wages for the period 2017-2020. Since 2016 is the first year with employment data, the first year with analysis of wage changes is from 2016 to 2017. The wage change analyses were restricted to employees who stayed in the same establishment between two adjacent years. The basic adjustments model (column 1) control for sex, marital status, number of children, age and age squared, seniority and seniority squared, year of observation, and municipality of residence, while the next two models successively add control for control for eight levels of educational attainment (column 2) and detailed educational level-and-field fixed effects (column 3). Adjusting for basic controls and detailed educational level-and-field fixed effects, the next three models separately control for occupation (column 4), establishment (column 5), and occupation-establishment (column 6) fixed effects. Huber-White heteroskedasticity robust standard errors are clustered on the person.

* P<0.05, ** P<0.01, *** P<0.001

Table A.7. Estimated immigrant--native gaps in logarithm of hourly wage for all employees (Panel A), logarithm of hourly wage for newly hired employees (Panel C), and annual change in logarithm of hourly wage for employees staying in the same establishment (Panel C) for immigrant-background workers from Western Europe and North America. OLS regressions.

				Within		
	Basic adjust-ments	Education, level	Education, level and field	Occupation	Establishment	Occupation-Establishment
	(1)	(2)	(3)	(4)	(5)	(6)
**Panel A. Log Hourly Wage of All Employees**						
Adult immigrants	-0.080***	-0.093***	-0.084***	-0.046***	-0.056***	-0.022***
	(0.000)	(0.000)	(0.000)	(0.000)	(0.000)	(0.000)
Childhood immigrants	-0.039***	-0.025***	-0.020***	-0.011***	-0.009***	-0.001
	(0.002)	(0.002)	(0.001)	(0.001)	(0.001)	(0.001)
Children of immigrants	-0.005	-0.014***	-0.010***	-0.008***	-0.006*	-0.006**
	(0.003)	(0.003)	(0.003)	(0.002)	(0.002)	(0.002)
R^2	0.252	0.408	0.452	0.613	0.615	0.768
Observations (person-years)	7,150,531	7,150,531	7,150,531	7,150,531	7,150,531	7,150,531
**Panel B. Log Hourly Wage of New Employees**						
Adult immigrants	-0.100***	-0.104***	-0.092***	-0.047***	-0.050***	-0.018***
	(0.001)	(0.001)	(0.001)	(0.001)	(0.001)	(0.001)
Childhood immigrants	-0.044***	-0.025***	-0.019***	-0.008**	-0.002	0.002
	(0.004)	(0.003)	(0.003)	(0.003)	(0.003)	(0.003)
Children of immigrants	-0.013†	-0.021**	-0.014*	-0.010*	-0.01	-0.006
	(0.007)	(0.007)	(0.006)	(0.005)	(0.006)	(0.006)
R^2	0.225	0.379	0.422	0.597	0.614	0.751
Observations (person-years)	1,040,687	1,040,687	1,040,687	1,040,687	1,040,687	1,040,687
**Panel C. Annual Change in Log Hourly Wage**						
Adult immigrants	0.001***	0.000	0.001*	0.001***	0.001	0.000
	(0.000)	(0.000)	(0.000)	(0.000)	(0.000)	(0.000)
Childhood immigrants	0.000	0.000	0.001	0.001	0.000	-0.001
	(0.001)	(0.001)	(0.001)	(0.001)	(0.001)	(0.001)
Children of immigrants	0.000	0.000	0.000	0.000	0.000	0.001
	(0.002)	(0.002)	(0.002)	(0.002)	(0.002)	(0.002)
R^2	0.011	0.012	0.013	0.017	0.114	0.118
Observations (person-years)	3,296,783	3,296,783	3,296,783	3,296,783	3,296,783	3,296,783

Note: Panel A reports estimates from OLS regressions where immigrant background was regressed on log hourly wages for the period 2016-2020. Panel B reports estimates from OLS regressions where immigrant background was regressed on log hourly wages for workers in the first year of employment in the current establishment in the period 2017-2020, since we do not observe workplace seniority in the year prior to 2016 for all workers. Panel C reports estimates from OLS regressions where immigrant background was regressed on the annual change in log hourly wages for the period 2017-2020. Since 2016 is the first year with employment data, the first year with analysis of wage changes is from 2016 to 2017. The wage change analyses were restricted to employees who stayed in the same establishment between two adjacent years. The basic adjustments model (column 1) control for sex, marital status, number of children, age and age squared, seniority and seniority squared, year of observation, and municipality of residence, while the next two models successively add control for control for eight levels of educational attainment (column 2) and detailed educational level-and-field fixed effects (column 3). Adjusting for basic controls and detailed educational level-and-field fixed effects, the next three models separately control for occupation (column 4), establishment (column 5), and occupation-establishment (column 6) fixed effects. Huber-White heteroskedasticity robust standard errors are clustered on the person.
